# Supplementary material for: Identification of photoperiod-induced specific miRNAs in the adrenal glands of Sunite sheep (Ovis aries)
Source: Front Vet Sci. 2022 Jul 22;9:888207. doi: 10.3389/fvets.2022.888207 (PMC9354845; doi:10.3389/fvets.2022.888207)
Supplement: Supplementary file 1 [file Data_Sheet_1.ZIP › Supplementary file/miRDeep2 file.zip!/miRDeep2 file/Know_Structure/LP42A2/oar-mir-181a-2.pdf]

miRBase precursor : oar-mir-181a-2
Total read count : 21655
oar-miR-181a read count : 21507
remaining reads : 148

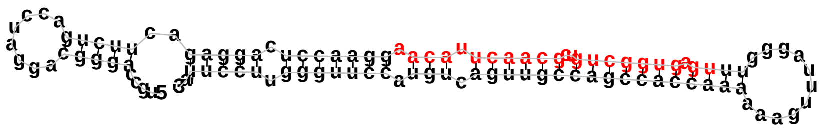

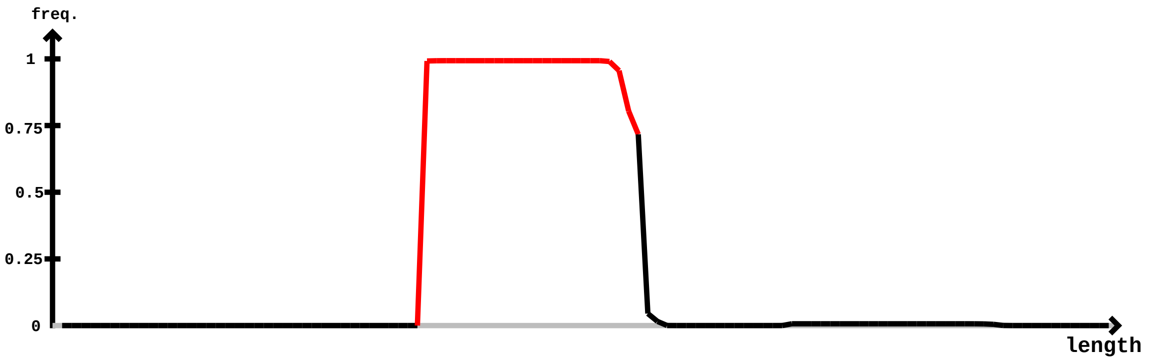

oar-miR-181a

5'- ugccagggcaggauccagucuucagaggacuccaaggaacauucaacgcugucggugaguuugggauuugaaaaaaccaccgaccguugacuguaccuuggguuccuua -3'	exp		
....(((((........)))))..(((((.(((((((.(((.((((((..(((((((.((((...........))))))))))))))))).))).))))))).))))).	reads	mm	sample
..................ucuucagaggacuccaagg..	1	0	seq
.....................................aacauucaacgcugucgg..	1	0	seq
.....................................aacauucaacgcugucggu...	64	0	seq
.....................................aacauucaacgcugucggug..	743	0	seq
.....................................aacauucaacgcugucgguga...	3282	0	seq
.....................................aacauucaacgcugucggugag..	1887	0	seq
.....................................aacauucaacgcugucggugagu...	14534	0	seq
.....................................aacauucaacgcugucggugaguu..	639	0	seq
.....................................aacauucaacgcugucggugaguuu...	330	0	seq
.....................................aacauucaacgcugucggugaguuug..	4	0	seq
.....................................aacauucaacgcugucggugaguuugggauuu..	1	0	seq
......................................acauucaacgcugucggugag..	1	0	seq
......................................acauucaacgcugucggugagu...	17	0	seq
......................................acauucaacgcugucggugaguuu...	1	0	seq
....cauucaacgcugucgguga...	1	0	seq
....cauucaacgcugucggugagu...	2	0	seq
....cauucaacgcugucggugaguuu...	1	0	seq
....aaccaccgaccguugacugua..............	1	0	seq
....aaccaccgaccguugacuguac.............	1	0	seq
....accaccgaccguugacugu...............	4	0	seq
....accaccgaccguugacugua..............	6	0	seq
....accaccgaccguugacuguac.............	34	0	seq
....accaccgaccguugacuguacc............	91	0	seq
....accaccgaccguugacuguaccu...........	9	0	seq
